# Using Gene Ontology to describe the role of the neurexin-neuroligin-SHANK complex in human, mouse and rat and its relevance to autism

**DOI:** 10.1186/s12859-015-0622-0

**Published:** 2015-06-06

**Authors:** Sejal Patel, Paola Roncaglia, Ruth C. Lovering

**Affiliations:** Institute of Medical Science, University of Toronto, Medical Sciences Building, 1 King’s College Circle, Toronto, M5S 1A8 Canada; Campbell Family Mental Health Research Institute, Centre for Addiction and Mental Health, 250 College Street, Toronto, M5T 1R8 Canada; Centre for Cardiovascular Genetics, Institute of Cardiovascular Science, University College London, Rayne Building, 5 University Street, London, WC1E 6JF UK; European Molecular Biology Laboratory, European Bioinformatics Institute (EMBL-EBI), Wellcome Trust Genome Campus, Hinxton, Cambridge, CB10 1SD UK; The Gene Ontology Consortium, http://geneontology.org/

**Keywords:** Gene Ontology, Autistic spectrum disorder, Synaptogenesis, Annotation, Neurexin, Neuroligin, SHANK, DLG4, GO

## Abstract

**Background:**

People with an autistic spectrum disorder (ASD) display a variety of characteristic behavioral traits, including impaired social interaction, communication difficulties and repetitive behavior. This complex neurodevelopment disorder is known to be associated with a combination of genetic and environmental factors. Neurexins and neuroligins play a key role in synaptogenesis and neurexin-neuroligin adhesion is one of several processes that have been implicated in autism spectrum disorders.

**Results:**

In this report we describe the manual annotation of a selection of gene products known to be associated with autism and/or the neurexin-neuroligin-SHANK complex and demonstrate how a focused annotation approach leads to the creation of more descriptive Gene Ontology (GO) terms, as well as an increase in both the number of gene product annotations and their granularity, thus improving the data available in the GO database.

**Conclusions:**

The manual annotations we describe will impact on the functional analysis of a variety of future autism-relevant datasets. Comprehensive gene annotation is an essential aspect of genomic and proteomic studies, as the quality of gene annotations incorporated into statistical analysis tools affects the effective interpretation of data obtained through genome wide association studies, next generation sequencing, proteomic and transcriptomic datasets.

**Electronic supplementary material:**

The online version of this article (doi:10.1186/s12859-015-0622-0) contains supplementary material, which is available to authorized users.

## Background

**Fig. 1:**
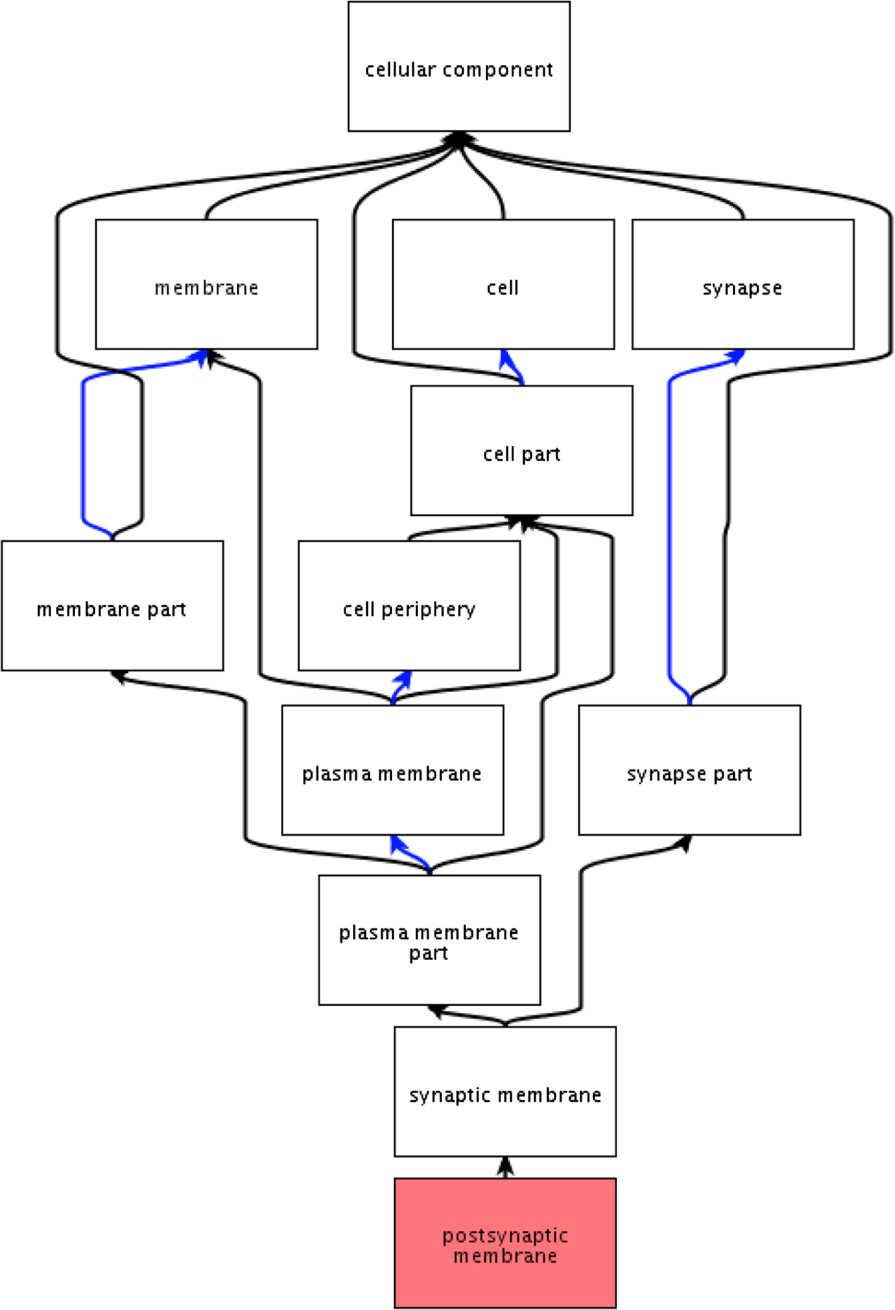
Part of the synapse domain of the GO *cellular component* ontology. The QuickGO (http://www.ebi.ac.uk/QuickGO) view of the parent–child relationships between the GO terms ‘*postsynaptic membrane*’ and ‘*cellular component*’. The black arrows are used where a term has an ‘is_a’ relationship to its parent term, the blue arrows indicate a ‘part_of’ relationship, www.geneontology.org/GO.ontology.relations.shtml

The Gene Ontology (GO; www.geneontology.org) contains controlled vocabulary terms (GO terms), which are connected through defined relationships within a hierarchical order (Fig. 1) [1]. The association of GO terms with gene products enables proteins to be classified (grouped) according to their shared normal *molecular functions*, the *biological processes* they contribute to, and their location with respect to subcellular compartments (*cellular components*). Summarising the known role of gene products from published papers to populate the GO database, a process known as annotation, allows researchers to have access to information on the role of individual proteins and protein families in the form of controlled vocabulary terms [2]. GO provides one of the major annotation resources used for the analysis of high-throughput datasets, such as those from transcriptomic and proteomic studies, to identify pathways, functions or cellular components over-represented within a dataset. For example, common GO domains found in an analysis of a brain transcriptomic dataset associated with aging in the prefrontal cortical regions were calcium signalling, protein tyrosine kinase signalling, electrical excitability and neuropeptide hormones [3]. In addition, GO is also being used in pathway-driven analysis tools to identify risk Single Nucleotide Polymorphisms (SNPs) associated with specific phenotypes, and to inform biomarker identification studies [4–6]. However, the interpretation of these datasets depends on the quality of the gene annotations available and the statistical analysis tools used, and we have previously demonstrated that cardiovascular-focused manual GO annotation significantly improves the interpretation of cardiovascular-relevant datasets [7].

Autistic spectrum disorder (ASD) is a complex neurodevelopmental condition known to be associated with a combination of genetic [8] and environmental factors [9]. ASD is often diagnosed around 3 years of age and many of the characteristic traits continue to adulthood [10]. Patients with ASD (as classified by the Diagnostic and Statistical Manual of Mental Disorders, Fifth Edition, DSM-5) [11] exhibit a variety of behavioral deficits, such as social interaction and communication difficulties and restrictive and repetitive behavior [12]. Limited social interaction by ASD children is often interpreted as a lack of social interest, but it is now recognised to be the inability of ASD children to engage with others [13–15]. ASD patients also have limited communication skills, such as gesture, facial expression and speech deficits. Repetitive stereotype behaviors are usually detailed and structure orientated, where the activity and/or behavior contains a number of steps in order to complete the task [13, 14].

There is strong evidence for multiple genetic factors determining the development of ASD. To date, around 40 genomic loci and over 100 genes have been associated with ASD based on rare variant approaches. These approaches have identified sequence and copy number variants as well as chromosomal rearrangements [16]. These genes and loci have been identified based on SNPs, *de novo* losses or gain mutations and linkage studies [17, 18]. Although many single genes have been implicated, a complete understanding of ASD is still to be achieved. A key obstacle for researchers is that these SNPs or mutations are not common in all patients with ASD, consequently the identification of low frequency SNPs associated with ASD is challenging.

Synaptogenesis, chromatin re-modeling, morphogenic developmental processes, calcium homeostasis and mitochondrial function have all been identified as potentially contributing to ASD [18]. In this study, we have focused on the manual annotation of the neurexin, neuroligin and SHANK protein families, as the interaction between the neurexin and neuroligin cell adhesion molecules is one of the processes that has been implicated in the development of ASD [18]. However, variants in the neurexin, neuroligin and *SHANK* gene families have only been associated with a few percent of ASD cases [18].

Neurexins (NRXNs) and neuroligins (NLGNs) are single-pass transmembrane proteins that play a key role in synaptogenesis, a process which occurs before birth and continues into adulthood [19]. In the process of synapse formation, the NLGN and NRXN cell surface adhesion molecules interact in a calcium-dependent manner to initiate the first stage of synapse formation and the assembly of protein components required for presynaptic and postsynaptic cell membrane organization [20, 21]. The intercellular interaction between NRXNs and NLGNs facilitates axo-dendritic contact [19, 21], and the synapse that is formed becomes stabilized or eliminated based on the synaptic activity, which is driven by the action potential between two or more neurons [22]. The scaffolding SHANK proteins are required for the proper formation and function of neuronal synapses and are connected to NLGN cell adhesion molecules via their interaction with DLG4 (PSD-95, postsynaptic density-95) to facilitate postsynaptic organization of cytoskeletal and signaling complexes [23].

ASD-associated variants have been identified in members of both the *NLGN* [24] and *NRXN* [25, 26] gene families. Although all five members of the *NLGN* gene family are associated directly with synapse assembly [27–29], mutations in only 3 members of this family, *NLGN3, NLGN4X* and *NLGN4Y,* have been identified in autistic patients [24, 30–32]. There are also five members of the *NRXN* gene family in humans: *NRXN1*, *NRXN2*, *NRXN3* [33] and two *NRXN4* genes known as *CNTNAP1* and *CNTNAP2* (Contactin-associated protein 1 and 2 respectively) [34]. *NRXN1-3* encode alpha and beta isoforms which have identical C-terminal transmembrane regions and cytoplasmic tails. However, alternative promoter use leads to the isoforms having different N-terminal extracellular sequences, the α-isoforms having much longer extracellular domains than the β-isoforms [29]. UniProt recognises these differences by providing unique identifiers for each isoform, rather than treating the isoforms as splice variants and listing the variants within a single protein record. Both the α- and β-isoforms have been associated with synapse formation [35], whereas the proteins encoded by *CNTNAP1* and *CNTNAP2* are involved in non-synaptic neurogenic processes [36, 37]*.* Mutations associated with ASD are found in both alpha and beta *NRXN1* [26, 38], *NRXN2* [39], *NRXN3* [40] and *CNTNAP2* [41]. Furthermore, variants in all three SHANK family members have been associated with ASD [42–44]. Neuroligin, neurexin and SHANK mutations link ASD with the molecular components of synaptogenesis. Therefore investigating the functional role of the proteins associated with the neurexin-neuroligin-SHANK complex in model organisms may explain how the mutations in some of these proteins result in the behavioral traits seen in some patients with ASD. DLG4 was also included in this focused annotation project due to its role as a scaffold protein connecting the NLGN and SHANK proteins [45].

Comprehensive manual annotation of the NRXN, NLGN, SHANK and DLG4 proteins identified several GO domains that are associated with the majority of these proteins, including behavior, synaptogenesis and neurogenesis. During the annotation process we identified a lack of GO terms which could describe the role of NRXNs and NLGNs in the process of synaptogenesis; to fill this void, new synapse assembly GO terms were created. These new GO terms were then associated with the NRXN, NLGN, SHANK and DLG4 proteins, when there was supporting experimental evidence. Furthermore, confirmed orthology predictions between the human, rat and mouse ASD-associated proteins supported the propagation of GO annotations from each protein to the orthologous proteins in these species (http://www.geneontology.org/cgi-bin/references.cgi#GO_REF:0000024) [2]. These new GO annotations and terms are now included in the GO database, enriching both the annotation data and the ontology structure.

## Methods

### Selection of experimental data to annotate

The PubMed database (http://www.ncbi.nlm.nih.gov/pubmed) was used to locate papers that contained experimental data describing each member of the NRXN, NLGN, SHANK or DLG4 protein families. The searches were performed, during April to November 2011, using the following gene names and symbols: neurexin, neuroligin, NRXN*, NLGN*, SHANK1, SHANK2, SHANK3, CASPR, DLG4, DLG-4, PSD95, PSD-95. This search retrieved over 2000 papers. Consequently, we undertook a PubMed search with each individual gene symbol or name and additional filters, in order to provide a comprehensive coverage of the role of these proteins with respect to autism. The following filters were applied to each of the symbol and name searches: ‘AND autism/ASD’, ‘AND synaptogenesis/synapse assembly’, ‘AND autism/ASD AND synaptogenesis/synapse assembly’. The number of papers available that described each gene influenced the number of filters applied. The decision about which paper to annotate was then based on whether: 1) new information would be added to the current GO annotation data associated with the protein; 2) it was possible to identify the species the protein or transfected cDNA construct was derived from. Only papers that met both criteria were annotated. The choice of papers annotated was therefore influenced by the information captured in previously annotated papers. In total, 66 papers with experimental data that were relevant to ASD or synaptogenesis were originally selected for annotation. However, following the identification of an association of NLGN4Y, NRXN2 and NRXN3 alpha and beta variants with ASD [39, 40], these two additional papers were also annotated (February 2014) bringing the total number of papers annotated to 68 (see Additional file 1).

### Identification of orthologous proteins

Orthologous proteins were identified for the NRXN, NLGN, SHANK and DLG4 protein families between the human, mouse and rat species, using the HUGO Gene Nomenclature Committee (http://www.genenames.org/) ortholog prediction tool (HCOP). HCOP compiles data from 11 different orthology prediction tools, including EnsemblCompara, Homologene and Inparanoid [46]. The predicted ortholog amino acid sequences were also aligned, using the default settings on the Basic Local Alignment Search Tool – BLASTP (http://blast.ncbi.nlm.nih.gov/Blast.cgi), to confirm high homology. In all cases there was greater than 89 % amino acid sequence identity between these aligned mammalian orthologs.

### Gene Ontology annotation - manual curation process

Manual GO annotation requires a GO curator to read publications and convert the data presented into an annotation. Each annotation includes the protein identifier, a GO term, an evidence code, and a reference [2]. During this focused annotation project GO terms were associated with protein records based on experimental data describing the human, rat and mouse NRXN, NLGN, SHANK and DLG4 proteins. The QuickGO browser (http://www.ebi.ac.uk/QuickGO) was used to identify the most specific GO terms to ‘capture’ the experimental data presented in each paper, and a consistent annotation approach was undertaken [2]. Evidence codes were associated to each annotation based on the type of experimental data presented in the paper (www.geneontology.org/GO.evidence.shtml) [2]. To complete the manual annotation process, GO annotations with experimental evidence codes were transferred to orthologous human, mouse and rat proteins. These annotations include the ISS (Inferred from Sequence Similarity) evidence code. New, more descriptive GO terms were created, along with improvements to the ontology structure, using the GO editorial tool, OBO-Edit [47].

## Results and discussion

### GO annotation of the neurexin, neuroligin, SHANK and DLG4 protein families

There are several thousand publications describing the mammalian members of the NRXN, NLGN, SHANK and DLG4 protein families. With limited resources available, we restricted our manual annotation focus to experimental data describing the functional role and cellular location of these proteins, where there was a clear relevance to synaptogenesis and autism. Consequently, only a fraction of the available experimental data describing the NRXN, NLGN, SHANK and DLG4 proteins has been captured. Synaptogenesis and other pathways that impact on behavior are complex processes, involving numerous proteins, and full annotation of these processes is not attempted in this report. GO terms were associated with the mammalian members of the NRXN, NLGN, SHANK and DLG4 protein families based on the experimental data present in 68 published experimental papers (see Additional files 1, 2 and 3), increasing the number of papers contributing to the manual annotations of these proteins from 172 to 240. Whenever possible, annotations were created that capture the *molecular function* of each protein, the *biological processes* these proteins contribute to, and their intracellular location (*cellular component*, see Additional file 4). This approach has created over 500 publication-supported manual annotations (see Additional files 3, 4, 5 and 6), doubling the previous number of these annotations to over 1000. In addition, to maximise the utility of this annotation project, almost 700 GO annotations were propagated to orthologs in the three species annotated (human, mouse and rat). These annotations are identified by the associated ISS (Inferred from Sequence Similarity) evidence code and were only created when orthology was confirmed and when the GO term was not already ‘manually’ associated with the protein record [2]. The propagation of annotations across these mammalian orthologs increased the number of manual annotations to these 47 proteins to over 1800 (see Additional file 3). There are now *molecular function*, *biological process* and *cellular component* annotations associated with almost all members of these families supported by confirmed experimental data. No experimental data was identified to support *cellular component* annotations for the human, mouse or rat NLGN4Y, NRXN2 and NRXN3 proteins, or to support *molecular function* annotations for the mouse or rat Nlgn4l proteins. This annotation approach has meant that over 400 unique GO manual annotations, directly supported by experimental evidence, are now associated with the human NRXN, NLGN, SHANK and DLG4 protein families. The BHF-UCL and UniProt-GOA teams have created the majority of manual annotations associated with the human NRXNs, NLGNs, SHANKs and DLG4 protein families (see Additional file 7).

The full definition and ontology placement for each GO term listed below is available in the QuickGO term page using the hyperlinks provided.

### Cellular component GO terms

**Fig. 2:**
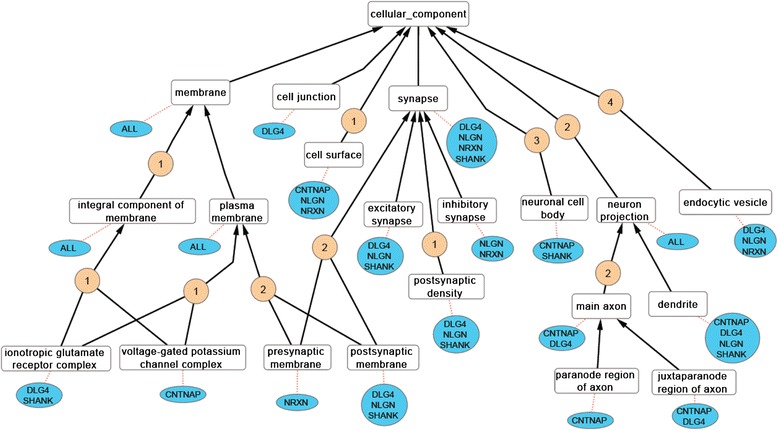
*Cellular component* GO terms associated manually to the ASD-relevant human proteins. A subsection of the *cellular component* domain, with the human protein families (blue ovals) and their associated GO terms (white boxes), linked with red dotted line (see Additional file 4). All associations (except ‘Inferred from Electronic Annotation’ (IEA) supported data) are included. If one or several member(s) of a protein family is associated with a specific term then the family name, or the protein name DLG4, is listed below the term. ‘ALL’ is used when a member of the CNTNAP, NLGN, NRXN, SHANK families as well as DLG4 have been associated with the GO term. The numbered circle indicates the minimum number of GO terms in the ancestor chart between two of the listed GO terms. For example there are 2 GO terms (‘*synaptic membrane*’ and ‘*synapse part*’) between the GO terms ‘*synapse*’ and ‘*postsynaptic membrane*’ (Fig. 1)

The manual annotation of 87 papers confirms the cellular location of many of the human, rat and mouse NRXN, NLGN, SHANK and DLG4 proteins (see Additional files 3 and 4). These *cellular component* GO annotations are primarily associated with either the evidence code IDA (Inferred from Direct Assay) or ISS. Furthermore, many of these *cellular component* manual annotations describe the neuron-relevant localisation of these proteins, such as ‘*excitatory synapse*’, ‘*postsynaptic membrane*’, and ‘*dendrite*’ (Fig. 2).

NRXNs and NLGNs contain a single transmembrane region, and their involvement in cell adhesion suggests they are located in the plasma membrane. Experimental evidence supports the association of the GO terms ‘*plasma membrane*’ and ‘*cell surface*’ with many of these proteins. However, in order to associate the GO term ‘*plasma membrane*’ with human NRXN1-α the evidence code IC (Inferred by Curator) is applied. This inference is based on the immunofluorescence analysis of NRXN1-α transfected COS cells [39], which supports the NRXN1-α ‘*cell surface*’ annotation, along with the knowledge that this protein contains a transmembrane domain and is therefore likely to be located in the plasma membrane.

The NRXN, NLGN, SHANK and DLG4 proteins play a key role in synapse assembly and there is considerable experimental evidence to support GO annotations describing the localization of these proteins to neuronal-specific components, such as ‘*excitatory synapse*’ [23, 48–52] and ‘*dendrite*’ [28, 51, 53, 54]. In addition, while the majority of the NRXN family are associated with the synapse, the CNTNAP proteins are located in the ‘*juxtaparanode region of axon*’ and ‘*paranode region of axon*’ [36, 37] (Fig. 2, see Additional files 3 and 4).

### Biological process GO terms

**Fig. 3:**
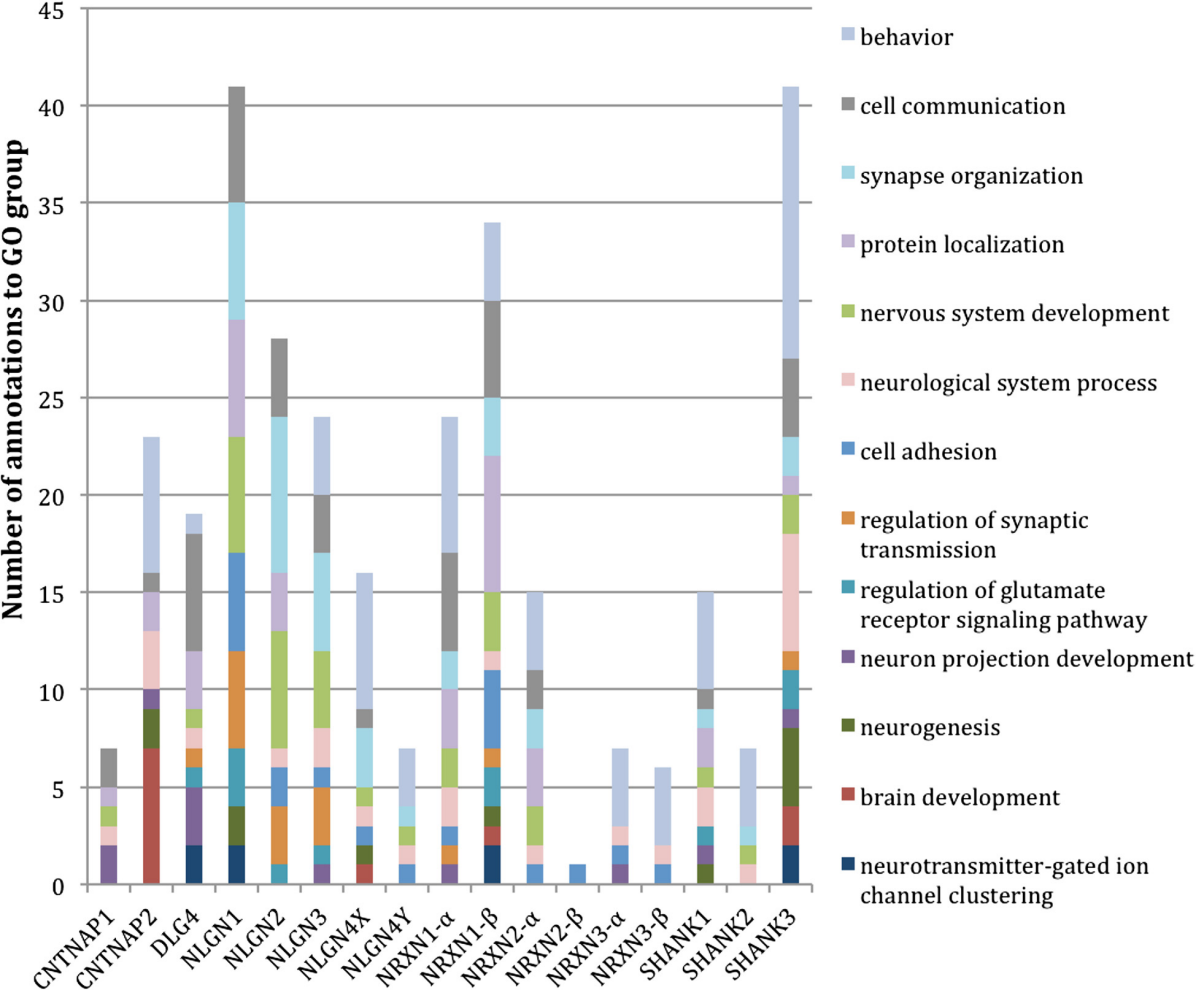
Distribution of *biological process* GO terms associated to each of the selected human protein records. The GO terms ‘directly’ associated with a human protein record have been grouped into broader *biological process* parent GO term categories (see Additional file 5). Due to the structure of the ontology there are some GO terms that are child terms of more than one of these broader GO parent terms. In these instances the annotation is then represented more than once in the histogram. No IEA annotations are included in the histogram

Several neurological processes are associated with the NRXN, NLGN, SHANK and DLG4 proteins families, such as neurogenesis, synaptic organisation, synaptic transmission, and behavior (Fig. 3). In contrast, CNTNAP1 and CNTNAP2 are involved in axon assembly, with CNTNAP1 required for neuronal action potential propagation [37, 55]. The *cellular component* and *biological process* annotations associated with the CNTNAPs reflect the lack of evidence for a role of these proteins in synaptic processes and identifies these proteins as functionally distinct from the other members of the NRXN family.

#### Synapse assembly

A synapse includes cellular components contributed by two adjacent cells. Consequently, synapse assembly covers a wide variety of processes, including presynaptic membrane assembly, postsynaptic membrane assembly, postsynaptic density assembly and the clustering of the various adhesion molecules, receptors, channels and scaffold proteins.

**Fig. 4:**
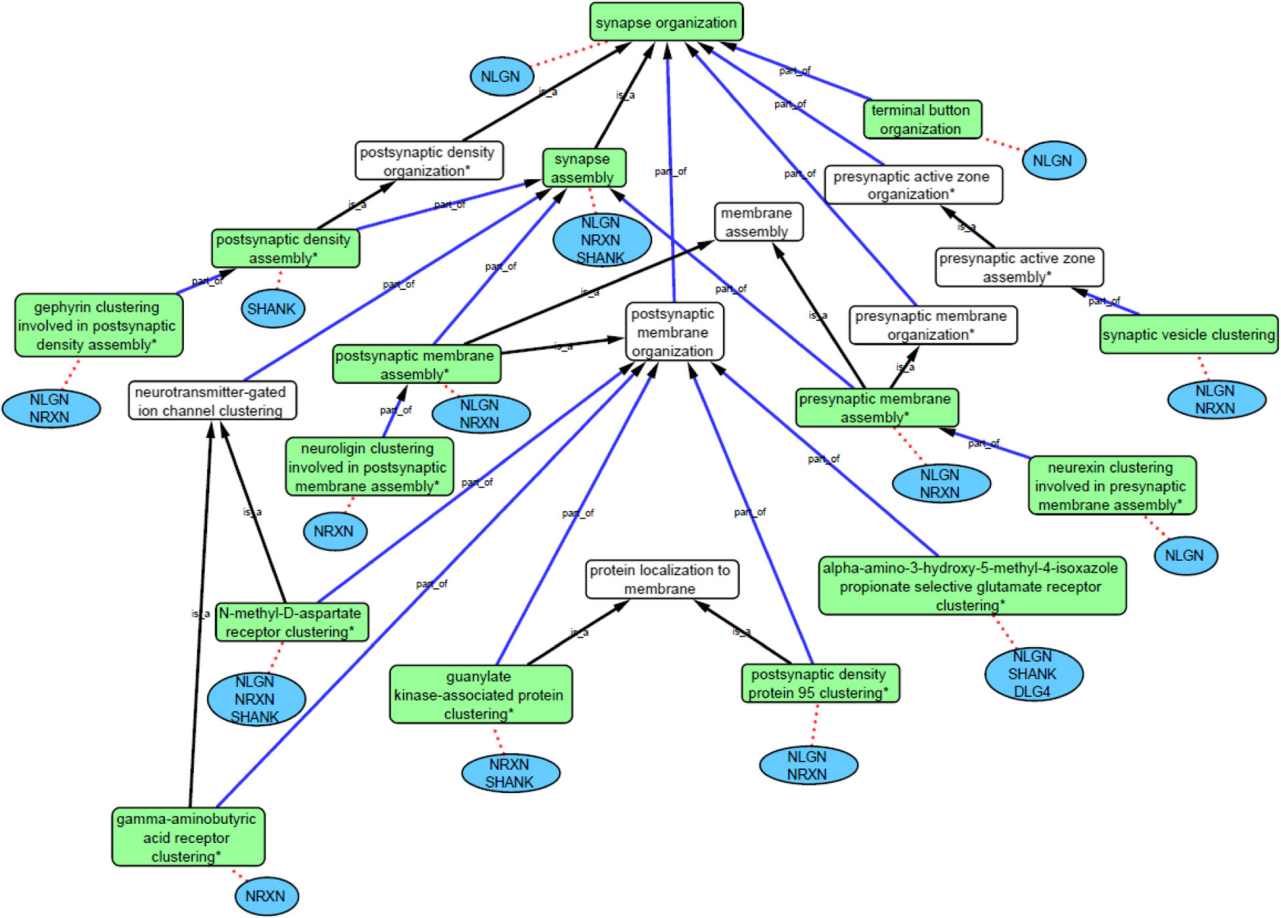
Ancestral relationships of the new GO terms created to support detailed ASD-relevant annotations in the domain of synapse organization. A subsection of the ‘*synapse organization*’ domain ontology, human protein families (blue ovals) and their associated GO terms (green boxes), linked with red dotted line (see Additional files 5 and 8); all associations (except IEA-supported data) are included. If one or several member(s) of a protein family is associated with a specific term then the family name, or the protein name DLG4, is listed below the term. * indicates new GO terms (Table 1). Black arrows indicate an ‘is_a’ relationship between a term and its parent; blue arrows indicate a ‘part_of’ relationship

The calcium-dependent interaction between an NLGN and NRXN protein, located in different cells, initiates the first stage of synapse formation [20, 21, 49, 56]. Therefore, NRXNs and NLGNs are annotated with ‘*calcium-dependent cell-cell adhesion via plasma membrane cell adhesion molecules*’ [35], ‘*heterophilic cell-cell adhesion via plasma membrane cell adhesion molecule*’ [35, 57]’ and ‘*neuron cell-cell adhesion*’ [29, 35, 56]. In addition, the NRXNs and NLGNs play an essential role in the clustering of proteins at the presynaptic membrane and postsynaptic membrane during synapse assembly [21, 35, 48]. In order to capture the specific detail of synapse assembly new, highly descriptive GO terms have been created (Table 1, Fig. 4). The assembly process involves organization of the synaptic membrane on either side of the synapse [35], consequently two new GO terms were created, *‘postsynaptic membrane assembly*’ and ‘*presynaptic membrane assembly*’ with ‘is_a’ child relationships to the *biological process* GO terms ‘*postsynaptic membrane organization*’ or ‘*presynaptic membrane organization*’, respectively. These new GO terms enabled the mouse Nrxn1-α, Nrxn1-β, Nrxn2-α and Nlgn1-3 [35, 39, 48, 58] to be annotated with *‘postsynaptic membrane assembly*’ based on experimental data. Similarly, the synaptic impact of murine Nlgn1 or Nlgn2 ectopic expression, in mixed culture assays, supports the association of ‘*presynaptic membrane assembly*’ [21, 35, 48]. Furthermore, to capture the involvement of NLGNs and NRXNs in initiating the clustering of specific proteins and organelles to synaptic locations, additional new GO terms have been created (Table 1, Fig. 4). Clustering of scaffold and receptor proteins is part of the process of postsynaptic and presynaptic membrane organization. Therefore, new GO terms, describing the clustering of these specific proteins, were created as ‘part_of’ child terms to either ‘*postsynaptic membrane organization*’ or ‘*presynaptic membrane organization*’ terms, with ‘is_a’ child relationships to ‘*protein localization to membrane*’, or part_of child terms to ‘*postsynaptic density assembly*’, with ‘is_a’ child relationships to ‘*protein localization to synapse*’. There is considerable experimental evidence describing the clustering of specific proteins at the synaptic membrane area. For example Gauthier et al., [39] showed that when Nrxn2-α is mutated there is impairment in GABAergic postsynaptic components and gephyrin at the dendrite contact sites of postsynaptic neurons [48]. Experimental data such as this has been used to support the association of the new GO term ‘*gephyrin clusteringinvolved in postsynaptic density assembly*’ with Nrxn1-α, Nrxn1-β and Nrxn2-α and the new GO term *‘postsynaptic density protein 95 clustering’* with Nrxn1-α, Nrxn1-β, Nrxn2-α, Ngln1 and Nlgn2 [35, 39, 48, 59] (Fig. 4).

**Table 1:** New GO terms created to support detailed ASD-relevant annotations. The ancestral relationship of these new GO terms is described in Figs. 4 and 5 (with the exception of the new protein binding terms and ‘*receptor localization to synapse*’). The term records in the QuickGO browser (http://www.ebi.ac.uk/QuickGO) provide the full definition and ontology placement for each GO term provide the full definition and ontology placement for each GO term. The reference column lists the references used as a source of information when creating each term

New GO Term	GO ID	Reference
Synapse assembly terms		
Postsynaptic density assembly	GO:0097107	[65]
Postsynaptic density organization	GO:0097106	[65]
Postsynaptic membrane assembly	GO:0097104	[39]
Presynaptic membrane assembly	GO:0097105	[56, 68]
Presynaptic membrane organization	GO:0097090	[49]
Regulation of postsynaptic membrane potential terms		
Negative regulation of excitatory postsynaptic membrane potential	GO:0090394	[49]
Positive regulation of excitatory postsynaptic membrane potential	GO:2000463	[77]
Positive regulation of inhibitory postsynaptic membrane potential	GO:0097151	[68]
Protein clustering/localization terms		
Alpha-amino-3-hydroxy-5-methyl-4-isoxazole propionate receptor clustering	GO:0097113	[35]
Gamma-aminobutyric acid receptor clustering	GO:0097112	[48]
Gephyrin clustering involved in postsynaptic density assembly	GO:0097116	[48]
Guanylate kinase-associated protein clustering	GO:0097117	[48]
Neurexin clustering involved in presynaptic membrane assembly	GO:0097115	[35]
Neuroligin clustering involved in postsynaptic membrane assembly	GO:0097118	[35]
N-methyl-D-aspartate receptor clustering	GO:0097114	[48]
Postsynaptic density protein 95 clustering	GO:0097119	[80]
Receptor localization to synapse	GO:0097120	[65]
Protein binding terms		
Neuroligin family protein binding	GO:0097109	[39]
Scaffold protein binding	GO:0097110	[80]

Following common practice when creating GO terms, the *cellular component* ontology was used to guide the *biological process* ontology. For example, the *cellular component* term ‘*postsynaptic density*’ ‘is_a’ child term of ‘*cytoskeletal part*’ and, therefore, in the *biological process* ontology ‘*postsynaptic density organization*’ has an ‘is_a’ relationship link with ‘*cytoskeleton organization*’. Biochemical analysis of postsynaptic densities purified from the striatum of wild type and *Shank3B*^*−*/*−*^ mice demonstrates that the Shank3 protein is required for correct postsynaptic density assembly [23]. Consequently, the new GO term ‘*postsynaptic density organization*’ is associated with murine Shank3.

Synaptic vesicle clustering occurs at the presynaptic membrane, as well as below this membrane. Therefore, the new GO term ‘*synaptic vesicle clustering*’ is placed as part_of ‘*presynaptic active zone assembly*’ rather than as a child term of ‘*presynaptic membrane organization*’. This ‘*synaptic vesicle clustering*’ GO term is associated with both murine Nlgn1 and Nrxn1-β, based on Nlgn1 ectopic expression data, or the recruitment of synaptic vesicle markers in cultured hippocampal neurons, following oligomerization of overexpressed murine Nrxn1-β, respectively [21, 35, 60].

The two closely related paralogs CNTNAP1 and CNTNAP2 are both associated with neurogenesis, but only CNTNAP2 has been found to be associated with ASD [61]. In contrast to the other members of the NRXN family, CNTNAP1 is found on paranode region of axons [36, 37], whereas CNTNAP2 is located on the juxtaparanode region of axons [36, 62]. Furthermore, CNTNAPs appear to play a role in axon assembly, rather than synapse assembly. For example, Garcia-Fresco *et al.* [37], show impaired localization of mitochondria and neurofilament to the paranodal region of the axon in mice deficient in Cntnap1. Based on this experimental evidence the GO term ‘*protein localization to paranode region of axon*’ is associated with murine Cntnap1 protein record (see Additional file 5) [55]. Furthermore, deletion of *Cntnap2* in mice demonstrates that Cntnap2 is required for clustering of voltage-gated potassium channels to the juxtaparanodal region of myelinated axons, similar to the role of NRXNs in neurotransmitter-gated ion channel clustering at the synapse [63]. This data was captured using the GO terms: ‘*clustering of voltage-gated potassium channels*’ and ‘*protein localization to juxtaparanode region of axon*’.

The synaptic scaffold SHANK family members and DLG4 are also key proteins in synapse assembly, providing essential structural support, and are involved in bringing necessary protein components to the synapse [48, 52]. Wang *et al*. [64] identifies that levels of Homer1b/c and GKAP in the postsynaptic density and GluA1 and NR2A in the synaptic plasma membrane are lower in *Shank3*^*e4–9*^ mice, compared to wild type mice. GluA1 is a subunit of the AMPA receptor; therefore, the GO term ‘*alpha-amino-3-hydroxy-5-methyl-4-isoxazole propionate selective receptor clustering*’ is associated with the mouse Shank3 protein record. This term is also applied to the rat Dlg4 protein, as RNA interference knockdown of rat Dlg4 in mixed culture assay leads to a reduction in the number of AMPA receptor type structures near the synapse compared to controls [65]. Further experimental evidence also supports the role of other NLGN, NRXN, SHANK and DLG4 proteins in the process of AMPA, GABA and NMDA receptor clustering in glutamatergic postsynaptic cells, and GO annotations capturing this information are now available [35, 48, 59, 64–67] (Fig. 4, see Additional files 5 and 8).

#### Regulation of postsynaptic membrane potential

**Fig. 5:**
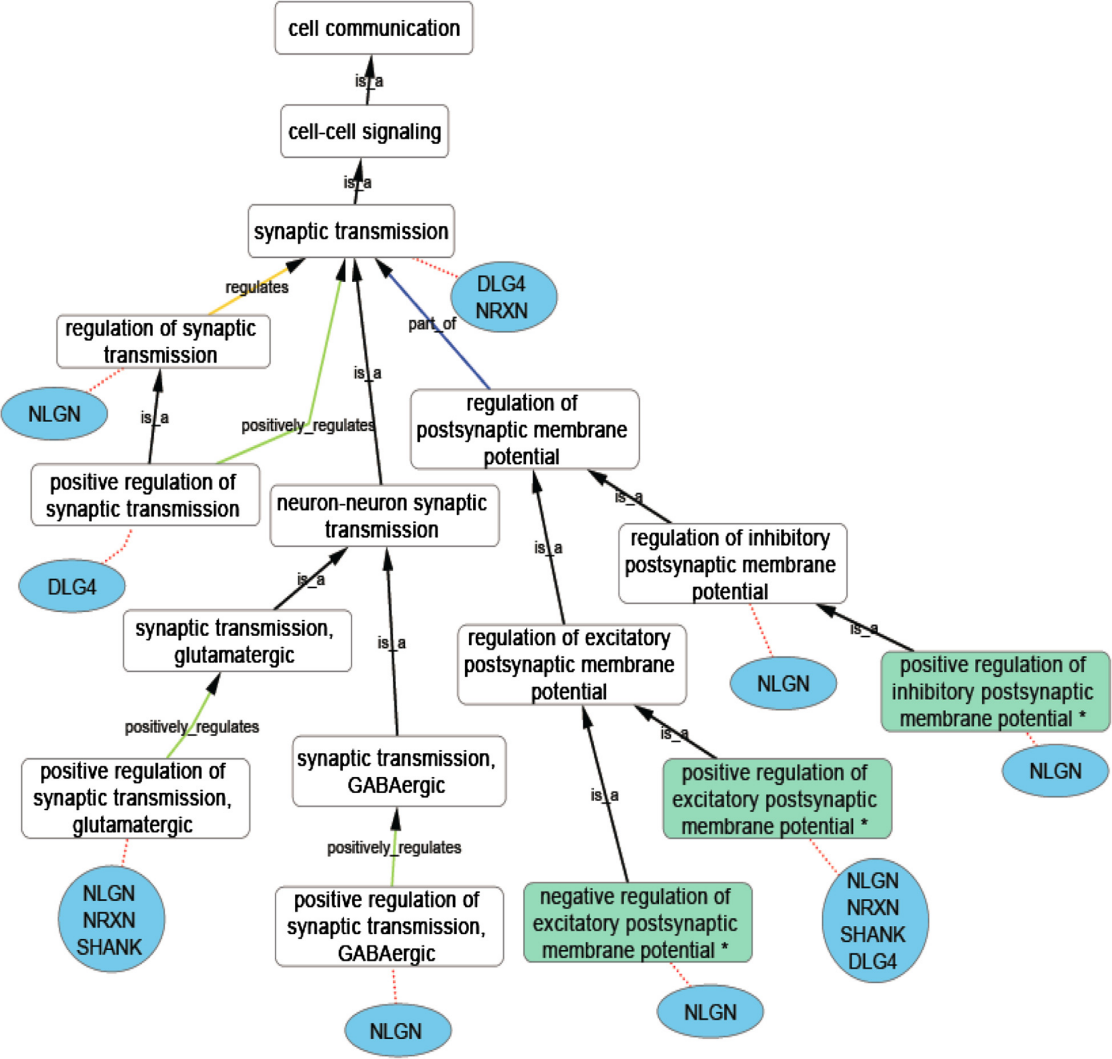
Ancestral relationships of the new GO terms created to support detailed ASD-relevant annotations in the domain of cell communication. A subsection of the ‘*cell communication*’ domain ontology, human protein families (blue ovals) and their associated GO terms (green boxes), linked with red dotted line (see Additional files 5 and 8); all associations (except IEA-supported data) are included. If one or several member(s) of a protein family is associated with a specific term then the family name, or the protein name DLG4, is listed below the term. * indicates new GO terms (Table 1). Black arrows indicate an ‘is_a’ relationship between a term and its parent; blue arrows, a ‘part_of’ relationship; yellow arrows, a ‘regulation’ relationship; green arrows, a ‘positive_regulation’ relationship

Experimental evidence supports the association of the GO term ‘*regulation of excitatory postsynaptic membrane potential*’ (or child terms) with the NLGN, NRXN, SHANK and DLG4 proteins [28, 51] (Fig. 5, see Additional files 5 and 8). For example, cultured neurons transfected with rat Nlgn1^(R473C)^, corresponding to a human variant associated with an autism disorder, showed a decrease in excitatory synaptic transmission for both AMPA receptor and NMDA receptor response, suggesting positive regulation of this process [51]. In contrast, neurons transfected with human NLGN4X selectively suppress the frequency of mEPSCs but not mIPSCs, suggesting that NLGN4X is involved in ‘*negative regulation of excitatory postsynaptic membrane potential*’ [28]. Transfection and transgenic data also supports the association of ‘*regulation of inhibitory postsynaptic membrane potential*’ (or child terms) with mouse and rat Nlgn2 and Nlgn3 proteins [48, 51, 68, 69] (Fig. 5, see Additional files 5 and 8)*.* Neurons transfected with rat Nlgn3^(R471C)^, corresponding to another ASD associated variant, have a decreased inhibitory postsynaptic membrane potential [69]. Although both Nlgn2 and Nlgn3 [50] are found within inhibitory and excitatory synapses, the expression of Nlgn2 and Nlgn3 is greater at the inhibitory synapse [48, 51, 69]. This difference of expression appears to be reflected in their role in the regulation of membrane potentials. The role of the NRXN and NLGN proteins in excitatory and inhibitory synaptic transmission suggests that a balance between these processes is necessary for normal brain development and that the dysregulation of these processes may be linked to the behavioral phenotypes seen in ASD individuals [19]. However, NRXN, NLGN, SHANK and DLG4 proteins also play a role in synaptic plasticity [70], which is also likely to contribute to the ASD phenotypes.

#### Social behavior

Impaired communication and social behavior are the key behavioral changes seen in ASD individuals, and variants in the *NRXN* [26, 38], *NLGN* [28, 71], *SHANK* [42–44] gene families as well as in the *CNTNAP2* gene are associated with ASD [72]. Consequently, using patient information provided by papers describing the association of variants in these genes with ASD, it was possible to apply several behavioral GO terms to these protein records, including ‘*social behavior*’, ‘*vocal learning*’ and ‘*adult behavior*’ (Table 2, see Additional files 5 and 8).

**Table 2:** *Biological process* behavioral GO terms associated with the ASD-relevant human and mouse proteins

	Human												Mouse							
GO term name	NLGN3	NLGN4X	NLGN4Y	NRXN1-α	NRXN1-β	NRXN2-α	NRXN3-α	NRXN3-β	CNTNAP2	SHANK1	SHANK2	SHANK3	Nlgn2	Nlgn3	Nlgn4l	Nrxn1-α	Dlg4	Shank1	Shank2	Shank3
adult behavior	X	X		X	X	X	X	X	X	X	X	X				X			X	
learning	X	X	X	X	X		X	X	X		X	X				X			X	X
social behavior	X	X	X	X	X	X	X	X	X	X	X	X		X	X		X	X	X	X
vocalization behavior	X	X	X	X	X	X	X	X	X	X	X	X			X		X	X	X	X
vocal learning				X		X			X			X								
locomotory exploration behavior													X				X			X
visual learning														X						
male courtship behavior															X					
territorial aggressive behavior															X					
regulation of grooming behavior																X	X			X
associative learning																		X		
habituation																		X		
long-term memory																		X		
olfactory behavior																		X		
memory																			X	X
exploration behavior																			X	
regulation of behavioral fear response																				X

X indicates the existence of an association between the GO term and the protein record, based on behavioral traits associated with variant genes only (IMP, Inferred from Mutant Phenotype, or IGI, Inferred from Genetic Interaction, supported annotations, see Additional files 5 and 8)

Model organisms have been used to investigate the impact of ASD-associated gene mutations on animal behavior. Simple behaviors in model organisms can provide information about the more complex behaviors seen in humans. For example, Hines *et al.* [68], measured social behavior in rats by recording how often a rat would choose to visit a room with another rat in it, compared to visiting a room without another rat in it. While a wild type rat would visit the occupied room more often than the empty room, a rat carrying an *Nlgn2* mutation displayed no preference between either rooms [68]. These types of rat and mouse behavioral studies were annotated, using the GO term ‘*social behavior*’*.* This simple behavior is similar to that observed in ASD individuals, where there is no eye contact or interest in another person in the room, and this ASD phenotype is also captured using the GO term ‘*social behavior*’ [24, 39, 44, 72].

Vocalization is also impaired in mice and rats carrying defects in *Shank* [73], *Nlgn4l* [74] or *Dlg4* [75] genes. For example, male *Shank3* heterozygous mice make fewer ultrasonic vocalizations to female mice, compared to their male wild type littermates [70]. This experiment supports the annotation ‘*vocalization behavior*’. Similarly, there is often an impairment of communication in ASD individuals with variants in the *NRXN1-α* [38]*, NRXN1-β* [26], *CNTNAP2* [41], *NLGN4X* [24, 28] and *SHANK1* [76] genes (Table 2). These ASD communication traits are captured either with the use of ‘*vocalization behavior*’ or more specifically ‘*vocal learning*’. Human proteins are only associated with the GO term ‘*vocal learning*’ when the authors provide detailed information about a lack of speech or very limited word usage [39, 43, 72] (see Additional files 3 and 5).

Behavior phenotypes described in mouse or rat cannot be mapped exactly to human behaviors. Furthermore, some of these behavioral traits are not relevant to human, such as ‘*male courtship behavior*’ and ‘*olfactory behavior*’. Consequently, very few behavioral annotations have been transferred from mouse or rat proteins to the human orthologs (Table 2, see Additional files 5 and 8). For example, behavioral studies support the association of the GO term ‘*regulation of grooming behavior*’ with the mouse Shank3, Dlg4 and Nrxn1-α proteins [23, 75, 77]. These annotations have been transferred, using the ISS evidence code, to the rat orthologs but not to the orthologous human proteins. Similarly the GO term ‘*exploration behavior*’ and its child term ‘*locomotory exploration behavior*’ are associated with the mouse Shank2 [54] and Shank3, Dlg4 and Nlgn2 [23, 75, 78] proteins (respectively), but not propagated to the human orthologs. In addition, we found no published evidence that variants in *DLG4* and *NLGN2* are associated with behavioral traits in human (Table 2, see Additional files 5 and 8).

### Molecular function GO terms

The majority of *molecular function* GO terms associated with the members of the *NRXN, NLGN, SHANK* and *DLG4* gene families capture information about the protein interactions they participate in (Table 3, see Additional file 6). To provide full annotation of the function of these proteins two new GO terms were created: ‘*neuroligin family protein binding*’ and ‘*scaffold protein binding*’ (Table 1). These new terms enable the interactions between the NRXN, NLGN, SHANK and DLG4 proteins to be described with the use of the GO terms ‘*neurexin family protein binding*’ [28, 35, 39, 71], ‘*neuroli gin family protein binding*’ [39, 79] and ‘*scaffold protein binding*’ [79–81] (Table 3, see Additional file 6). The *molecular function* annotations were also used to identify the specific classes of receptors bound by the SHANK and DLG4 proteins, such as ‘*ionotropic glutamate receptor binding*’ [53], ‘*beta-1 adrenergic receptor binding*’ [82] and ‘*P2Y1 nucleotide receptor binding*’ [83]. In addition, the NRXN and NLGN proteins are associated with ‘*receptor activity*’ [35, 39, 56, 68], whereas the SHANK proteins are associated with the GO term ‘*GKAP/Homer scaffold activity*’ [48, 52, 80] (Table 3, see Additional file 6).

**Table 3:** Selection of the *molecular function* GO terms associated with the ASD-relevant human proteins

GO term name	NLGN1	NLGN2	NLGN3	NLGN4X	NLGN4Y	NRXN1-α	NRXN1-β	NRXN2-α	NRXN2-β	NRXN3-α	NRXN3-β	CNTNAP1	SHANK1	SHANK2	SHANK3	DLG4
cell adhesion molecule binding	X	X	X	X	X	X	X	X	X	X	X					
receptor activity	X	X	X	X	X	X	X	X	X	X	X	X				
neurexin family protein binding	X	X	X	X	X											
neuroligin family protein binding						X	X	X	X	X	X					X
calcium channel regulator activity						X	X	X		X						
acetylcholine receptor binding						X										X
GKAP/Homer scaffold activity													X	X	X	
scaffold protein binding			X	X	X								X		X	X
receptor signaling complex scaffold activity													X			
ionotropic glutamate receptor binding													X	X	X	X
somatostatin receptor binding													X			
beta-1 adrenergic receptor binding																X
D1 dopamine receptor binding																X
P2Y1 nucleotide receptor binding																X

X indicates the existence of an association between the GO term and the human protein record; all associations (except IEA-supported data) are included (see Additional file 6)

### Impact of focused annotation approaches on data interpretation

Members of the GO Consortium have undertaken a variety of focused manual annotation approaches to annotate the human proteome. Two large manual annotation efforts include renal and cardiovascular focuses [7, 84], while other projects have focused on specific cellular components, *e.g.* the peroxisome [85], or specific individual genes, *e.g.* those annotated by the Reference Genome Project [86]. It is possible that these focused annotation approaches could lead to a bias in the human annotation data, which could impact on the analysis of high-throughput datasets. However, to date, there is no evidence of unexpected cardiovascular, renal, peroxisome or neurological terms being detected in term enrichment analyses [7, 84]. Furthermore, during the manual annotation of these 4 protein families, 68 additional genes were annotated based on the evidence presented in these 68 papers, reducing any potential arising bias.

Since their creation, the 19 new GO terms have been associated with 58 distinct human proteins creating 158 annotations (see Additional file 9); of these, only 31 annotations are based on experimental data, the majority of the remainder have been created through the transfer of annotations dependent on orthology assertions. Fifty-two of these annotations capture protein binding interactions whereas the remaining 106 are associated with the new synaptogenesis related terms. In addition to the NRXN, NLGN, SHANK and DLG4 proteins, 27 other proteins, APOE, ATP2B4, CELF4, CEP112, CHRNA7, DRD4, GRID2, GRIN1, HOMER1, IKBKB, IL1RAPL1, LRP4, MAP3K7, MDM2, MTMR2, NLGN4X, NOS1, NPY2R, P2RX7, P2RY1, PANX1, PRKCZ, PTEN, PTK2B, PTPRD, RELN, S1PR2, SCN5A, are annotated to these new biological process GO terms. All of the new GO terms presented in this paper, apart from scaffold protein binding, are directly relevant to synaptogenesis in the context of ASD, demonstrating the impairment in the NGLN-NRXN-SHANK complex. Future annotation of proteins involved in synaptogenesis may provide a useful approach to explore and identify other ASD risk candidates. All of the new GO terms presented here were created in August 2011 or earlier, and yet the majority of 104 manual annotations to human proteins applying these GO terms (in March 2014) were created during this focused annotation project (91 annotations). For highly specific annotations to be created by manual GO curation the curators need to feel confident in the biological field they are annotating. Curators working within a specific annotation project improve their understanding of a biological area and improve their knowledge of the GO terms available to describe the experiments they are annotating. The high number of annotations using these new GO terms created by this focused project, compared to the number created by other groups, highlights the importance of focused annotation approaches to comprehensive annotation of the human genome. However, annotation projects that target the annotation of large number of proteins, such as the UniProt-GO annotation project [87], ensure the breadth of annotation is maintained and reduce the bias within the GO database.

Several groups have now used functional annotation data to identify candidate risk alleles associated with complex multigenic diseases [88, 89]. Continued annotation of neurological processes, as well as other ASD-relevant processes such as chromatin re-modeling, developmental processes, calcium homeostasis and mitochondrial function, and the application of pathway-based analysis statistical approaches may, therefore, help with the identification of additional ASD risk alleles within genome-wide association studies and next generation sequencing projects.

## Conclusion

The annotation data and ontology terms within the GO database have been improved through this focused annotation project. Published experimental and patient data was used to capture the involvement of the NRXN, NLGN, SHANK and DLG4 proteins in synaptogenesis, neurogenesis and the behavioral traits seen in ASD. In order to create descriptive annotations the representation of synaptogenesis in GO was expanded, with the addition of 14 expressive terms within the synapse organization domain (Table 1, Fig. 4, 5). These new GO terms describe the more specific aspects of the synapse complex assembly, such as ‘*N-methyl-D-aspartate receptor clustering*’, ‘*neurexin clustering involved in presynaptic membrane assembly*’ and ‘*presynaptic membrane assembly*’, and enable a detailed description of the biological role of the NRXN, NLGN, DLG4 and SHANK proteins in synaptogenesis (see Additional files 5 and 8). Further work on the ontology is still needed to improve the description of synaptic processes using GO terms. Moreover, additional annotation projects would enable the comprehensive annotation of all ASD-relevant proteins, as well as, full annotation of neurological processes such as synaptic plasticity, synaptic organization and synaptic transmission.

GO is a dynamic database that is always expanding as new annotations are added and new GO terms are created in the ontology. The advantage of a focused annotation approach is that it ensures the immediate use of newly created GO terms for annotations (see Additional file 9). In contrast, GO terms created during the annotation of unrelated proteins may end up applied to only a few proteins, for a considerable time. The main challenge in annotating autism-relevant proteins was finding detailed experimental evidence for each protein. For example, despite extensive literature review, human NRXN1-β, NRXN2-α, NRXN2-β, NRXN3-α, and NRXN3-β have no experimental evidence code supported GO *molecular function* terms annotations. Furthermore, there are limited terms available in the behavioral domain of GO, and the Neurobehavior Ontology [90] may be better suited to provide a more comprehensive interpretation of complex behavioral traits than can be achieved with GO.

Variants in the *NRXN*, *NLGN* and *SHANK* gene families, and in the *DLG4* gene, have the potential to result in impaired synaptic formation and impaired regulation of synaptic transmission; however, not all of these proteins have been associated with ASD [19]. The quality of gene annotations incorporated into statistical analysis tools has a direct impact on the effective interpretation of many genomic and proteomic datasets. Unfortunately, not all functional analysis tools include current annotation data; some tools use annotation datasets that are over a year old. Additional ontology development and the continued comprehensive annotation of the proteins involved in ASD-relevant processes, capturing more data as it emerges in the literature, would ensure the maximum utility of the GO data for interpretation of ASD-focused transcriptomic, proteomic, genome-wide association studies and next generation sequencing.

## Additional files

Additional file 1:
**References used to support the annotation of NLGN, NRXN, SHANK and PSD-95 proteins with GO terms, as part of the ASD focused annotation project.**
Additional file 2: Table S1. List of NRXN, NLGN, SHANK and DLG4 proteins annotated with GO terms, relevant to ASD. Includes hyperlinks to the QuickGO browser listing current annotations associated with these proteins. Additional file 3: Table S2. Summary of the number of GO annotations associated with the NRXN, NLGN, SHANK and DLG4 human, mouse and rat protein families. The summary tab lists the number of GO annotations associated with the NRXN, NLGN, SHANK and DLG4 human, mouse and rat protein families. The all_data tab includes all annotations associated with these proteins. Downloaded using the QuickGO browser, filtering on the 47 protein IDs (see Additional file 2), 28 March 2014. Pre-focus annotations are those generated before the start of this autism-focused annotation project; Focus annotations are those generated by this autism-focused annotation project; Other annotations are those generated by other databases not involved in this autism-focused annotation project (*e.g.* MGI, RGD, UniProt); Automatic annotations are those generated by a variety of automatic scripts based on orthology (Ensembl), protein domain structure (InterPro) or UniProt Keyword mapping files (UniProt). Additional file 4: Table S3.
*Cellular component* GO annotations associated with the NRXN, NLGN, SHANK and DLG4 human, mouse and rat protein families. Downloaded using the QuickGO browser, filtering on the 47 protein IDs (see Additional file 2) and the GO ID GO:0005575, 28 March 2014. An additional column is provided entitled Pre-focus/Focus/Other/Automatic: Pre-focus annotations are those generated before the start of this autism-focused annotation project; Focus annotations are those generated by this autism-focused annotation project; Other annotations are those generated by other databases after the start of this autism-focused annotation project (*e.g.* MGI, RGD, UniProt); Automatic annotations are those generated by a variety of automatic scripts based on orthology (Ensembl), protein domain structure (InterPro) or UniProt Keyword mapping files (UniProt). Additional file 5: Table S4.
*Biological process* GO annotations associated with the NRXN, NLGN, SHANK and DLG4 human, mouse and rat protein families. The ‘direct’ GO term annotations are the GO terms that have been associated with the protein record. These direct GO terms have been grouped into broader *biological process* ‘grouping’ parent GO terms, or categories. Due to the structure of the ontology there are some direct GO terms that are child terms of more than one of these broader GO grouping parent terms. In these instances the annotation is then represented more than once in the dataset. Downloaded using the QuickGO browser, filtering on the 47 protein IDs (see Additional file 2) and the GO ID GO:0008150, 28 March 2014. An additional column is provided entitled Pre-focus/Focus/Other/Automatic: Pre-focus annotations are those generated before the start of this autism-focused annotation project; Focus annotations are those generated by this autism-focused annotation project; Other annotations are those generated by other databases after the start of this autism-focused annotation project (*e.g.* MGI, RGD, UniProt); Automatic annotations are those generated by a variety of automatic scripts based on orthology (Ensembl), protein domain structure (InterPro) or UniProt Keyword mapping files (UniProt). Additional file 6: Table S5.
*Molecular function* GO annotations associated with the NRXN, NLGN, SHANK and DLG4 human, mouse and rat protein families. Downloaded using the QuickGO browser, filtering on the 47 protein IDs (see Additional file 2) and the GO ID GO:0003674, 28 March 2014. An additional column is provided entitled Pre-focus/Focus/Other/Automatic: Pre-focus annotations are those generated before the start of this autism-focused annotation project; Focus annotations are those generated by this autism-focused annotation project; Other annotations are those generated by other databases after the start of this autism-focused annotation project (*e.g.* MGI, RGD, UniProt); Automatic annotations are those generated by a variety of automatic scripts based on orthology (Ensembl), protein domain structure (InterPro) or UniProt Keyword mapping files (UniProt). Additional file 7: Table S6. QuickGO statistics for the source of GO annotations associated with the NRXN, NLGN, SHANK and DLG4 human, mouse and rat protein families. Downloaded using the QuickGO browser, filtering on the 47 protein IDs (see Additional file 2), 28 March 2014. Additional file 8: Table S7. Summary table of the *biological process* GO annotations associated with human DLG4 and the human NRXN, NLGN and SHANK protein families. All annotations to the human protein records are included. The ‘direct’ GO term annotations are the GO terms that have been associated with the protein record (white rows). These direct GO terms have been grouped into broader *biological process* ‘grouping’ parent GO terms, or categories (green rows). Due to the structure of the ontology there are some direct GO terms that are child terms of more than one of these broader GO grouping parent terms. In these instances the annotation is then represented more than once in the dataset. Downloaded using the QuickGO browser, filtering on the 17 human protein IDs (see Additional file 2) and the GO ID GO:0008150, 28 March 2014. Additional file 9: Table S8. Number of annotations associated with new GO terms created to support detailed ASD-relevant annotations. Downloaded using the QuickGO browser, filtering on the new GO IDs (Table 1), 11 July 2014. Focus annotations are those generated by this autism-focused annotation project; Other annotations are those generated by other databases not involved in this autism-focused annotation project (*e.g.* MGI, RGD, UniProt); Automatic annotations are those generated by a variety of automatic scripts based on orthology (Ensembl), protein domain structure (InterPro) or UniProt Keyword mapping files (UniProt). For cases when multiple protein IDs are mapped to a single gene, the manual annotations are counted as a single annotation not multiple annotations, *e.g.* MGI has associated Lrcc4 with postsynaptic density protein 95 clustering [91], based on evidence presented in Kim S *et al*., 2006, this annotation is mapped to 4 protein IDs (E9PUX8, Q149E6, Q8BJ09, Q99PH1) in QuickGO; this is counted as a single annotation.

## Abbreviations

ASD: Autistic spectrum disorder
BLAST: Basic Local Alignment Search Tool
GO: Gene Ontology
HCOP: HUGO Gene Nomenclature Committee ortholog prediction tool
IC: Inferred by Curator
IDA: Inferred from Direct Assay
IEA: Inferred from Electronic Annotation
IGI: Inferred from Genetic Interaction
IMP: Inferred from Mutant Phenotype
ISS: Inferred from Sequence Similarity
MGI: Mouse Genome Informatics
NRXN: Neurexin
NLGN: Neuroligin
RGD: Rat Genome Database
SNP: Single Nucleotide Polymorphism
