# Supplementary material for: Using Gene Ontology to describe the role of the neurexin-neuroligin-SHANK complex in human, mouse and rat and its relevance to autism
Source: BMC Bioinformatics. 2015 Jun 6;16(1):186. doi: 10.1186/s12859-015-0622-0 (PMC4458007; doi:10.1186/s12859-015-0622-0)
Supplement: Additional file 1: — References used to support the annotation of NLGN, NRXN, SHANK and PSD-95 proteins with GO terms, as part of the ASD focused annotation project. [file 12859_2015_622_MOESM1_ESM.doc]

Alarcon M, Abrahams BS, Stone JL, Duvall JA, Perederiy JV, Bomar JM, Sebat J, Wigler M, Martin CL, Ledbetter DH*, et al*: **Linkage, association, and gene-expression analyses identify CNTNAP2 as an autism-susceptibility gene.** *American journal of human genetics* 2008, **82:**150-159. PMID:18179893

Bakkaloglu B, O'Roak BJ, Louvi A, Gupta AR, Abelson JF, Morgan TM, Chawarska K, Klin A, Ercan-Sencicek AG, Stillman AA*, et al*: **Molecular cytogenetic analysis and resequencing of contactin associated protein-like 2 in autism spectrum disorders.** *American journal of human genetics* 2008, **82:**165-173. PMID:18179895

Barrow SL, Constable JR, Clark E, El-Sabeawy F, McAllister AK, Washbourne P: **Neuroligin1: a cell adhesion molecule that recruits PSD-95 and NMDA receptors by distinct mechanisms during synaptogenesis.** *Neural development* 2009, **4:**17. PMID:19450252

Bel C, Oguievetskaia K, Pitaval C, Goutebroze L, Faivre-Sarrailh C: **Axonal targeting of Caspr2 in hippocampal neurons via selective somatodendritic endocytosis.** *Journal of cell science* 2009, **122:**3403-3413. PMID:19706678

Berkel S, Marshall CR, Weiss B, Howe J, Roeth R, Moog U, Endris V, Roberts W, Szatmari P, Pinto D*, et al*: **Mutations in the SHANK2 synaptic scaffolding gene in autism spectrum disorder and mental retardation.** *Nature genetics* 2010, **42:**489-491. PMID:20473310

Blundell J, Tabuchi K, Bolliger MF, Blaiss CA, Brose N, Liu X, Sudhof TC, Powell CM: **Increased anxiety-like behavior in mice lacking the inhibitory synapse cell adhesion molecule neuroligin 2.** *Genes, brain, and behavior* 2009, **8:**114-126. PMID:19016888

Bozdagi O, Sakurai T, Papapetrou D, Wang X, Dickstein DL, Takahashi N, Kajiwara Y, Yang M, Katz AM, Scattoni ML*, et al*: **Haploinsufficiency of the autism-associated Shank3 gene leads to deficits in synaptic function, social interaction, and social communication.** *Molecular autism* 2010, **1:**15. PMID:21167025

Chen SX, Tari PK, She K, Haas K: **Neurexin-neuroligin cell adhesion complexes contribute to synaptotropic dendritogenesis via growth stabilization mechanisms in vivo.** *Neuron* 2010, **67:**967-983. PMID:20869594

Chen X, Nelson CD, Li X, Winters CA, Azzam R, Sousa AA, Leapman RD, Gainer H, Sheng M, Reese TS: **PSD-95 is required to sustain the molecular organization of the postsynaptic density.** *The Journal of neuroscience : the official journal of the Society for Neuroscience* 2011, **31:**6329-6338. PMID:21525273

Cho KO, Hunt CA, Kennedy MB: **The rat brain postsynaptic density fraction contains a homolog of the Drosophila discs-large tumor suppressor protein.** *Neuron* 1992, **9:**929-942. PMID:1419001

Chubykin AA, Atasoy D, Etherton MR, Brose N, Kavalali ET, Gibson JR, Sudhof TC: **Activity-dependent validation of excitatory versus inhibitory synapses by neuroligin-1 versus neuroligin-2.** *Neuron* 2007, **54:**919-931. PMID:17582332

Chubykin AA, Liu X, Comoletti D, Tsigelny I, Taylor P, Sudhof TC: **Dissection of synapse induction by neuroligins: effect of a neuroligin mutation associated with autism.** *The Journal of biological chemistry* 2005, **280:**22365-22374. PMID:15797875

Comoletti D, De Jaco A, Jennings LL, Flynn RE, Gaietta G, Tsigelny I, Ellisman MH, Taylor P: **The Arg451Cys-neuroligin-3 mutation associated with autism reveals a defect in protein processing.** *The Journal of neuroscience : the official journal of the Society for Neuroscience* 2004, **24:**4889-4893. PMID:15152050

Dean C, Scholl FG, Choih J, DeMaria S, Berger J, Isacoff E, Scheiffele P: **Neurexin mediates the assembly of presynaptic terminals.** *Nature neuroscience* 2003, **6:**708-716. PMID:12796785

Durand CM, Betancur C, Boeckers TM, Bockmann J, Chaste P, Fauchereau F, Nygren G, Rastam M, Gillberg IC, Anckarsater H*, et al*: **Mutations in the gene encoding the synaptic scaffolding protein SHANK3 are associated with autism spectrum disorders.** *Nature genetics* 2007, **39:**25-27. PMID:17173049

Etherton M, Foldy C, Sharma M, Tabuchi K, Liu X, Shamloo M, Malenka RC, Sudhof TC: **Autism-linked neuroligin-3 R451C mutation differentially alters hippocampal and cortical synaptic function.** *Proceedings of the National Academy of Sciences of the United States of America* 2011, **108:**13764-13769. PMID:21808020

Etherton MR, Blaiss CA, Powell CM, Sudhof TC: **Mouse neurexin-1alpha deletion causes correlated electrophysiological and behavioral changes consistent with cognitive impairments.** *Proceedings of the National Academy of Sciences of the United States of America* 2009, **106:**17998-18003. PMID:19822762

Etherton MR, Tabuchi K, Sharma M, Ko J, Sudhof TC: **An autism-associated point mutation in the neuroligin cytoplasmic tail selectively impairs AMPA receptor-mediated synaptic transmission in hippocampus.** *The EMBO journal* 2011, **30:**2908-2919. PMID:21642956

Feng J, Schroer R, Yan J, Song W, Yang C, Bockholt A, Cook EH, Jr., Skinner C, Schwartz CE, Sommer SS: **High frequency of neurexin 1beta signal peptide structural variants in patients with autism.** *Neuroscience letters* 2006, **409:**10-13. PMID:17034946

Feyder M, Karlsson RM, Mathur P, Lyman M, Bock R, Momenan R, Munasinghe J, Scattoni ML, Ihne J, Camp M*, et al*: **Association of mouse Dlg4 (PSD-95) gene deletion and human DLG4 gene variation with phenotypes relevant to autism spectrum disorders and Williams' syndrome.** *The American journal of psychiatry* 2010, **167:**1508-1517. PMID:20952458

Fu Z, Washbourne P, Ortinski P, Vicini S: **Functional excitatory synapses in HEK293 cells expressing neuroligin and glutamate receptors.** *Journal of neurophysiology* 2003, **90:**3950-3957. PMID:12930820

Garcia-Fresco GP, Sousa AD, Pillai AM, Moy SS, Crawley JN, Tessarollo L, Dupree JL, Bhat MA: **Disruption of axo-glial junctions causes cytoskeletal disorganization and degeneration of Purkinje neuron axons.** *Proceedings of the National Academy of Sciences of the United States of America* 2006, **103:**5137-5142. PMID:16551741

Gauthier J, Siddiqui TJ, Huashan P, Yokomaku D, Hamdan FF, Champagne N, Lapointe M, Spiegelman D, Noreau A, Lafreniere RG*, et al*: **Truncating mutations in NRXN2 and NRXN1 in autism spectrum disorders and schizophrenia.** *Human genetics* 2011, **130:**563-573. PMID:21424692

Graf ER, Zhang X, Jin SX, Linhoff MW, Craig AM: **Neurexins induce differentiation of GABA and glutamate postsynaptic specializations via neuroligins.** *Cell* 2004, **119:**1013-1026. PMID:15620359

Gutierrez RC, Hung J, Zhang Y, Kertesz AC, Espina FJ, Colicos MA: **Altered synchrony and connectivity in neuronal networks expressing an autism-related mutation of neuroligin 3.** *Neuroscience* 2009, **162:**208-221. PMID:19406211

Heine M, Thoumine O, Mondin M, Tessier B, Giannone G, Choquet D: **Activity-independent and subunit-specific recruitment of functional AMPA receptors at neurexin/neuroligin contacts.** *Proceedings of the National Academy of Sciences of the United States of America* 2008, **105:**20947-20952. PMID:19098102

Hines RM, Wu L, Hines DJ, Steenland H, Mansour S, Dahlhaus R, Singaraja RR, Cao X, Sammler E, Hormuzdi SG*, et al*: **Synaptic imbalance, stereotypies, and impaired social interactions in mice with altered neuroligin 2 expression.** *The Journal of neuroscience : the official journal of the Society for Neuroscience* 2008, **28:**6055-6067. PMID:18550748

Hung AY, Futai K, Sala C, Valtschanoff JG, Ryu J, Woodworth MA, Kidd FL, Sung CC, Miyakawa T, Bear MF*, et al*: **Smaller dendritic spines, weaker synaptic transmission, but enhanced spatial learning in mice lacking Shank1.** *The Journal of neuroscience : the official journal of the Society for Neuroscience* 2008, **28:**1697-1708. PMID:18272690

Jamain S, Quach H, Betancur C, Rastam M, Colineaux C, Gillberg IC, Soderstrom H, Giros B, Leboyer M, Gillberg C, Bourgeron T: **Mutations of the X-linked genes encoding neuroligins NLGN3 and NLGN4 are associated with autism.** *Nature genetics* 2003, **34:**27-29. PMID:12669065

Jamain S, Radyushkin K, Hammerschmidt K, Granon S, Boretius S, Varoqueaux F, Ramanantsoa N, Gallego J, Ronnenberg A, Winter D*, et al*: **Reduced social interaction and ultrasonic communication in a mouse model of monogenic heritable autism.** *Proceedings of the National Academy of Sciences of the United States of America* 2008, **105:**1710-1715. PMID:18227507

Kim JE, O'Sullivan ML, Sanchez CA, Hwang M, Israel MA, Brennand K, Deerinck TJ, Goldstein LS, Gage FH, Ellisman MH, Ghosh A: **Investigating synapse formation and function using human pluripotent stem cell-derived neurons.** *Proceedings of the National Academy of Sciences of the United States of America* 2011, **108:**3005-3010. PMID:21278334

Kim JH, Yang E, Park JH, Yu YS, Kim KW: **Shank 2 expression coincides with neuronal differentiation in the developing retina.** *Experimental & molecular medicine* 2009, **41:**236-242. PMID:19299912

Ko J, Zhang C, Arac D, Boucard AA, Brunger AT, Sudhof TC: **Neuroligin-1 performs neurexin-dependent and neurexin-independent functions in synapse validation.** *The EMBO journal* 2009, **28:**3244-3255. PMID:19730411

Lee HW, Kim Y, Han K, Kim H, Kim E: **The phosphoinositide 3-phosphatase MTMR2 interacts with PSD-95 and maintains excitatory synapses by modulating endosomal traffic.** *The Journal of neuroscience : the official journal of the Society for Neuroscience* 2010, **30:**5508-5518. PMID:20410104

Levinson JN, Li R, Kang R, Moukhles H, El-Husseini A, Bamji SX: **Postsynaptic scaffolding molecules modulate the localization of neuroligins.** *Neuroscience* 2010, **165:**782-793. PMID:19914352

Mondin M, Labrousse V, Hosy E, Heine M, Tessier B, Levet F, Poujol C, Blanchet C, Choquet D, Thoumine O: **Neurexin-neuroligin adhesions capture surface-diffusing AMPA receptors through PSD-95 scaffolds.** *The Journal of neuroscience : the official journal of the Society for Neuroscience* 2011, **31:**13500-13515. PMID:21940442

Oiso S, Takeda Y, Futagawa T, Miura T, Kuchiiwa S, Nishida K, Ikeda R, Kariyazono H, Watanabe K, Yamada K: **Contactin-associated protein (Caspr) 2 interacts with carboxypeptidase E in the CNS.** *Journal of neurochemistry* 2009, **109:**158-167. PMID:19166515

Peca J, Feliciano C, Ting JT, Wang W, Wells MF, Venkatraman TN, Lascola CD, Fu Z, Feng G: **Shank3 mutant mice display autistic-like behaviours and striatal dysfunction.** *Nature* 2011, **472:**437-442. PMID:21423165

Pettem KL, Yokomaku D, Luo L, Linhoff MW, Prasad T, Connor SA, Siddiqui TJ, Kawabe H, Chen F, Zhang L*, et al*: **The specific alpha-neurexin interactor calsyntenin-3 promotes excitatory and inhibitory synapse development.** *Neuron* 2013, **80:**113-128. PMID:24094106

Poliak S, Gollan L, Martinez R, Custer A, Einheber S, Salzer JL, Trimmer JS, Shrager P, Peles E: **Caspr2, a new member of the neurexin superfamily, is localized at the juxtaparanodes of myelinated axons and associates with K+ channels.** *Neuron* 1999, **24:**1037-1047. PMID:10624965

Poliak S, Salomon D, Elhanany H, Sabanay H, Kiernan B, Pevny L, Stewart CL, Xu X, Chiu SY, Shrager P*, et al*: **Juxtaparanodal clustering of Shaker-like K+ channels in myelinated axons depends on Caspr2 and TAG-1.** *The Journal of cell biology* 2003, **162:**1149-1160. PMID:12963709

Romorini S, Piccoli G, Jiang M, Grossano P, Tonna N, Passafaro M, Zhang M, Sala C: **A functional role of postsynaptic density-95-guanylate kinase-associated protein complex in regulating Shank assembly and stability to synapses.** *The Journal of neuroscience : the official journal of the Society for Neuroscience* 2004, **24:**9391-9404. PMID:15496675

Saavedra MV, Smalla KH, Thomas U, Sandoval S, Olavarria K, Castillo K, Delgado MG, Delgado R, Gundelfinger ED, Bacigalupo J, Wyneken U: **Scaffolding proteins in highly purified rat olfactory cilia membranes.** *Neuroreport* 2008, **19:**1123-1126. PMID:18596612

Sato D, Lionel AC, Leblond CS, Prasad A, Pinto D, Walker S, O'Connor I, Russell C, Drmic IE, Hamdan FF*, et al*: **SHANK1 Deletions in Males with Autism Spectrum Disorder.** *American journal of human genetics* 2012, **90:**879-887. PMID:22503632

Scheiffele P, Fan J, Choih J, Fetter R, Serafini T: **Neuroligin expressed in nonneuronal cells triggers presynaptic development in contacting axons.** *Cell* 2000, **101:**657-669. PMID:10892652

Silverman JL, Turner SM, Barkan CL, Tolu SS, Saxena R, Hung AY, Sheng M, Crawley JN: **Sociability and motor functions in Shank1 mutant mice.** *Brain research* 2011, **1380:**120-137. PMID:20868654

Siow NL, Choi RC, Xie HQ, Kong LW, Chu GK, Chan GK, Simon J, Barnard EA, Tsim KW: **ATP induces synaptic gene expressions in cortical neurons: transduction and transcription control via P2Y1 receptors.** *Molecular pharmacology* 2010, **78:**1059-1071. PMID:20847060

Sudhof TC: **Neuroligins and neurexins link synaptic function to cognitive disease.** *Nature* 2008, **455:**903-911. PMID:18923512

Sun XY, Takagishi Y, Okabe E, Chishima Y, Kanou Y, Murase S, Mizumura K, Inaba M, Komatsu Y, Hayashi Y*, et al*: **A novel Caspr mutation causes the shambling mouse phenotype by disrupting axoglial interactions of myelinated nerves.** *Journal of neuropathology and experimental neurology* 2009, **68:**1207-1218. PMID:19816196

Traka M, Goutebroze L, Denisenko N, Bessa M, Nifli A, Havaki S, Iwakura Y, Fukamauchi F, Watanabe K, Soliven B*, et al*: **Association of TAG-1 with Caspr2 is essential for the molecular organization of juxtaparanodal regions of myelinated fibers.** *The Journal of cell biology* 2003, **162:**1161-1172. PMID:12975355

Tu JC, Xiao B, Naisbitt S, Yuan JP, Petralia RS, Brakeman P, Doan A, Aakalu VK, Lanahan AA, Sheng M, Worley PF: **Coupling of mGluR/Homer and PSD-95 complexes by the Shank family of postsynaptic density proteins.** *Neuron* 1999, **23:**583-592. PMID:10433269

Uchino S, Wada H, Honda S, Nakamura Y, Ondo Y, Uchiyama T, Tsutsumi M, Suzuki E, Hirasawa T, Kohsaka S: **Direct interaction of post-synaptic density-95/Dlg/ZO-1 domain-containing synaptic molecule Shank3 with GluR1 alpha-amino-3-hydroxy-5-methyl-4-isoxazole propionic acid receptor.** *Journal of neurochemistry* 2006, **97:**1203-1214. PMID:16606358

Uemura T, Lee SJ, Yasumura M, Takeuchi T, Yoshida T, Ra M, Taguchi R, Sakimura K, Mishina M: **Trans-synaptic interaction of GluRdelta2 and Neurexin through Cbln1 mediates synapse formation in the cerebellum.** *Cell* 2010, **141:**1068-1079. PMID:20537373

Uemura T, Mori H, Mishina M: **Direct interaction of GluRdelta2 with Shank scaffold proteins in cerebellar Purkinje cells.** *Molecular and cellular neurosciences* 2004, **26:**330-341. PMID:15207857

Vaags AK, Lionel AC, Sato D, Goodenberger M, Stein QP, Curran S, Ogilvie C, Ahn JW, Drmic I, Senman L*, et al*: **Rare deletions at the neurexin 3 locus in autism spectrum disorder.** *American journal of human genetics* 2012, **90:**133-141. PMID:22209245

Varoqueaux F, Aramuni G, Rawson RL, Mohrmann R, Missler M, Gottmann K, Zhang W, Sudhof TC, Brose N: **Neuroligins determine synapse maturation and function.** *Neuron* 2006, **51:**741-754. PMID:16982420

Vernes SC, Newbury DF, Abrahams BS, Winchester L, Nicod J, Groszer M, Alarcon M, Oliver PL, Davies KE, Geschwind DH*, et al*: **A functional genetic link between distinct developmental language disorders.** *The New England journal of medicine* 2008, **359:**2337-2345. PMID:18987363

Verpelli C, Dvoretskova E, Vicidomini C, Rossi F, Chiappalone M, Schoen M, Di Stefano B, Mantegazza R, Broccoli V, Bockers TM*, et al*: **Importance of Shank3 protein in regulating metabotropic glutamate receptor 5 (mGluR5) expression and signaling at synapses.** *The Journal of biological chemistry* 2011, **286:**34839-34850. PMID:21795692

Waga C, Okamoto N, Ondo Y, Fukumura-Kato R, Goto Y, Kohsaka S, Uchino S: **Novel variants of the SHANK3 gene in Japanese autistic patients with severe delayed speech development.** *Psychiatric genetics* 2011, **21:**208-211. PMID:21378602

Wang X, McCoy PA, Rodriguiz RM, Pan Y, Je HS, Roberts AC, Kim CJ, Berrios J, Colvin JS, Bousquet-Moore D*, et al*: **Synaptic dysfunction and abnormal behaviors in mice lacking major isoforms of Shank3.** *Human molecular genetics* 2011, **20:**3093-3108. PMID:21558424

Wittenmayer N, Korber C, Liu H, Kremer T, Varoqueaux F, Chapman ER, Brose N, Kuner T, Dresbach T: **Postsynaptic Neuroligin1 regulates presynaptic maturation.** *Proceedings of the National Academy of Sciences of the United States of America* 2009, **106:**13564-13569. PMID:19628693

Wohr M, Roullet FI, Hung AY, Sheng M, Crawley JN: **Communication impairments in mice lacking Shank1: reduced levels of ultrasonic vocalizations and scent marking behavior.** *PloS one* 2011, **6:**e20631. PMID:21695253

Won H, Lee HR, Gee HY, Mah W, Kim JI, Lee J, Ha S, Chung C, Jung ES, Cho YS*, et al*: **Autistic-like social behaviour in Shank2-mutant mice improved by restoring NMDA receptor function.** *Nature* 2012, **486:**261-265. PMID:22699620

Yan J, Feng J, Schroer R, Li W, Skinner C, Schwartz CE, Cook EH, Jr., Sommer SS: **Analysis of the neuroligin 4Y gene in patients with autism.** *Psychiatric genetics* 2008, **18:**204-207. PMID:18628683

Yoshida T, Yasumura M, Uemura T, Lee SJ, Ra M, Taguchi R, Iwakura Y, Mishina M: **IL-1 receptor accessory protein-like 1 associated with mental retardation and autism mediates synapse formation by trans-synaptic interaction with protein tyrosine phosphatase delta.** *The Journal of neuroscience : the official journal of the Society for Neuroscience* 2011, **31:**13485-13499. PMID:21940441

Zahir FR, Baross A, Delaney AD, Eydoux P, Fernandes ND, Pugh T, Marra MA, Friedman JM: **A patient with vertebral, cognitive and behavioural abnormalities and a de novo deletion of NRXN1alpha.** *Journal of medical genetics* 2008, **45:**239-243. PMID:18057082

Zhang C, Milunsky JM, Newton S, Ko J, Zhao G, Maher TA, Tager-Flusberg H, Bolliger MF, Carter AS, Boucard AA*, et al*: **A neuroligin-4 missense mutation associated with autism impairs neuroligin-4 folding and endoplasmic reticulum export.** *The Journal of neuroscience : the official journal of the Society for Neuroscience* 2009, **29:**10843-10854. PMID:19726642

Zweier C, de Jong EK, Zweier M, Orrico A, Ousager LB, Collins AL, Bijlsma EK, Oortveld MA, Ekici AB, Reis A*, et al*: **CNTNAP2 and NRXN1 are mutated in autosomal-recessive Pitt-Hopkins-like mental retardation and determine the level of a common synaptic protein in Drosophila.** *American journal of human genetics* 2009, **85:**655-666. PMID:19896112
